# Effect of different assumptions for brain water content on absolute measures of cerebral oxygenation determined by frequency-domain near-infrared spectroscopy in preterm infants: an observational study

**DOI:** 10.1186/1471-2431-14-206

**Published:** 2014-08-19

**Authors:** Anja Demel, Martin Wolf, Christian F Poets, Axel R Franz

**Affiliations:** 1 Department of Neonatology, University Children’s Hospital Tuebingen, Tuebingen, Germany; 2 Biomedical Optics Research Laboratory, Division of Neonatology, University Hospital Zurich, Zurich, Switzerland

**Keywords:** Brain water content, Regional cerebral oxygen saturation, Near-infrared spectroscopy, Preterm infant

## Abstract

**Background:**

Brain-water content (BWC) decreases with maturation of the brain and potentially affects parameters of cerebral oxygenation determined by near-infrared spectroscopy (NIRS). Most commercially available devices do not take these maturational changes into account. The aim of this study was to determine the effect of different assumptions for BWC on parameters of cerebral oxygenation in preterm infants.

**Methods:**

Concentrations of oxy-, deoxy- and total hemoglobin and regional cerebral oxygen saturation (rcStO_2_) were calculated based on absolute coefficients of absorption and scattering determined by multi-distance Frequency-Domain-NIRS assuming BWCs of 75-95%, which may be encountered in newborn infants depending on gestational and postnatal age.

**Results:**

This range of BWC gave rise to a linear modification of the assessed NIRS parameters with a maximum change of 10%. This may result in an absolute overestimation of rcStO_2_ by (median (range)) 4 (1–8)%, if the calculation is based on the lowest BWC (75%) in an extremely preterm infant with an anticipated BWC of 95%.

**Conclusion:**

Clinicians wishing to rely on parameters of cerebral oxygenation determined by NIRS should consider that maturational changes in BWC not taken into account by most devices may result in a deviation of cerebral oxygenation readings by up to 8% from the correct value.

## Background

Near-infrared spectroscopy (NIRS) is a tool to non-invasively evaluate tissue oxygenation in term and preterm infants. As reported previously [1-3], there are several instruments commercially available which use different techniques to measure tissue oxygenation, e.g. in the brain.

NIRS quantifies the interaction of near-infrared photons with biological tissue, which can be described by two different properties: the light absorption and the reduced scattering coefficient (μ_a_ and μ_s_’). Since deoxy- and oxyhemoglobin (HHb, O_2_Hb) are the most relevant chromophores absorbing light of the near-infrared spectrum their concentrations can be calculated from μ_a_. NIRS is able to determine μ_a_ by the diffusion approximation or changes in μ_a_ by the modified law of Lambert and Beer if the optical path length and geometrical properties are known. An important factor in these calculations is the brain water content (BWC) because water also absorbs near-infrared light, although to a lesser extent than O_2_Hb and HHb.

As far as the underlying algorithms for the determination of measures of tissue oxygenation are revealed at all, most NIRS devices do not allow adjustment for maturational changes in BWC and their underlying algorithms may assume BWCs as low as 75%.

Considering that due to physiological maturation BWC varies from 75% to 95% [4-6], one has to expect that this maturational change in BWC will have an effect on readings of cerebral oxygenation determined by NIRS, which might be therapeutically relevant in the clinical setting.

We aimed to quantify the impact of different assumptions for brain water content (75% for adults, 85% for term infants and 95% for very preterm infants [6]) in a series of measurements of cerebral oxygenation in preterm infants.

## Methods

This prospective observational study was approved by the ethics committee of Tuebingen University Hospital and written informed parental consent was obtained in all infants.

### Study population

A convenience sample of 17 preterm infants was studied in the neonatal intensive and high dependency care units of Tuebingen University Children’s Hospital. Patient characteristics are shown in Table 1. Only infants who were hemodynamically stable, i.e. who had normal blood pressure and normal skin colour and capillary refill time without cardiocirculatory support were included. Those with chromosomal or syndromal abnormalities were excluded.

**Table 1:** Demographic Characteristics of Infants Studied

**Clinical characteristics**	**Median/N**	**Range**
Preterm infants (n)	17	
Gender F/M (n/n)	8/9	
Gestational age at birth (weeks)	34 3/7	32 1/7 - 35 5/7
Postmenstrual age at measurement (weeks)	34 4/7	32 2/7 - 35 6/7
Postnatal age at measurement (days)	2	2
Birth weight (kg)	2.120	1.150 – 2.922
Head circumference (cm) at measurement	31.0	27.0 - 34.0
APGAR 5 min	8	8 – 10
Umbilical artery pH	7.30	7.20 - 7.40

### NIRS-Measurements

All measurements were performed with the infant sleeping in a supine position with the head slightly elevated and turned to the contralateral side by less than 30°. The probe was positioned at the right temporo-parietal-region accurately in the middle between the tragus and the sagittal suture to avoid the sagittal sinus and the Sylvian fissure. Care was taken to comb any existing newborn hair apart before placing the optode. The optode was applied to the infants skull held by the hand of the examiner with gentle pressure.

For each measurement, a recording lasting at least 2 minutes was performed at a sampling rate of 1Hz.

### NIRS-Device

We employed the ISS Oxiplex TS (ISS Inc., Champaign, IL, USA), a frequency-domain near infrared spectroscope. Each channel is equipped with 8 near-infrared light sources at two different wavelengths (four emitting at 692 nm and four at 834 nm) with emitter-detector distances of 1.5, 2, 2.5 and 3 cm, enabling a tissue penetration of 2–3 cm in depth according to the manufacturer’s specifications. To enable assessment of the path length as measured by a phase-shift, the light intensity is modulated with a frequency of 110 MHz.

Light intensity and phase shift are recorded for each emitter-optode distance using the proprietary software package OxiTS, and the absolute μ_a_ and μ_s_’ are calculated based on the slope of the respective regression lines at each wavelength using the diffusion equation for homogeneous, semi-infinite media [1,2]. Based on μ_a_ at two wavelengths, absolute concentrations of HHb and O_2_Hb and consequently also absolute values for total hemoglobin (tHb) and rcStO_2_ are calculated.

### Calculation of hemoglobin concentrations and hemoglobin oxygen saturation for different water contents

Calculations of O_2_Hb, HHb, tHb and rcStO_2_ were performed using the equations given below and assuming BWCs of 95%, 85% and 75% [6].

$$\begin{array}{l} \text{Computation}\;\text{of}\;\text{hemoglobin}\;\text{concentrations}\;\text{and}\;\text{measurement}\;\text{of}\;\text{tissue}\;\text{oxygenation}:\\ O_{2}\text{Hb}=1000\;*\left[aO_{2}Hb_{834}*\left(µa_{692}-E_{H2O\left(692\right)}*\text{WC}\right)\right)\\ \;\left(+aO_{2}Hb_{692}*\left(µa_{834}-E_{H2O\left(834\right)}*\text{WC}\right)\right]\\ \text{HHb}=1000*\left[\text{aHH}b_{834}*\left(µa_{692}-E_{H2O\left(692\right)}*\text{WC}\right)\right)\\ \;\left(+\text{aHH}b_{692}*\left(µa_{834}-E_{H2O\left(834\right)}*\text{WC}\right)\right]\\ \text{tHb}=O_{2}\text{Hb}+\text{HHb}\\ \text{rcSt}O_{2}=100*\frac{O_{2}\text{Hb}}{\text{tHb}}\;\\ E_{O2\text{Hb}\left(692\right)}=0.9556;\;E_{O2\text{Hb}\left(834\right)}=2.3670;\;\\ E_{\text{HHb}\left(692\right)}=4.7000;\;E_{\text{HHb}\left(834\right)}=1.7890\\ E_{H2O\left(834\right)}=0.00033637848;\;E_{H2O\left(692\right)}=0.00005606308\\ \text{Det}=E_{\text{HHb}\left(834\right)}\;*\;E_{O2\text{Hb}\left(692\right)}-E_{O2\text{Hb}\left(834\right)}*E_{\text{HHb}\left(692\right)}\\ \text{aHH}b_{834}=\frac{E_{O2\text{Hb}\left(692\right)}}{\text{Det}}\\ \text{aHH}b_{692}=\frac{-E_{O2\text{Hb}\left(834\right)}}{\text{Det}}\\ aO_{2}Hb_{834}=\frac{-E_{\text{HHb}\left(692\right)}}{\text{Det}}\\ aO_{2}Hb_{692}=\frac{E_{\text{HHb}\left(834\right)}}{\text{Det}}\;\\ \begin{array}{l} \begin{array}{l} \text{Legend}:\\ \text{Concentration}\;\text{of}: \end{array}\\ O_{2}\text{Hb}\;=\;\text{oxygenated}\;\text{hemoglobin}\;\left(µM\right);\;\\ \text{HHb}\;=\;\text{deoxygenated}\;\text{hemoglobin}\;\left(µM\right);\\ \text{tHb}\;=\;\text{total}\;\text{hemoglobin}\;\left(µM\right).\\ \text{rcSt}O_{2}=\;\text{regional}\;\text{cerebral}\;\text{oxygen}\;\text{saturation}\\ µa\;692\;/\;834\;=\;\text{absorption}\;\text{coefficient}\;\left(1/\text{cm}\right)\\ µ\mathrm{s’}\;692\;/\;834\;=\;\text{reduced}\;\text{scattering}\;\text{coefficient}\;\left(1/\text{cm}\right)\\ \text{WC}\;=\;\text{water}\;\text{content}\;\left(\%\right)\\ E_{H2O}=\;\text{extinction}\;\text{coefficient}\;\text{of}\;\text{water}\;=\;1/\;\left(\%\;*\;\text{mM}\right)\\ E_{\text{HHb}\left(692/834\right)}=\;\text{extinction}\;\text{coefficient}\;\text{of}\;\text{DeOxy}\;\text{hemoglobin}\;\\ \;\text{at}\;\text{wavelengt}h_{692/834}=\;1/\;\left(\%\;*\;\text{mM}\right)\\ E_{O2\text{Hb}\left(692/834\right)}=\;\text{extinction}\;\text{coefficient}\;\text{of}\;\text{Oxy}\;\text{hemoglobin}\;\\ \;\text{at}\;\text{wavelengt}h_{692/834}=\;1/\;\left(\%\;*\;\text{mM}\right) \end{array} \end{array}$$

### Data analysis

For each 2-min measurement the median of HHb, O_2_Hb, tHb and rcStO_2_ was calculated three times, assuming BWCs of 75, 85 and 95%, respectively. Data are depicted as median (minimum – maximum) of the 17 individual medians for each parameter and each assumption for BWC.

Differences in NIRS-parameters brought about by the different assumptions for BWC were evaluated for normal distribution using a Shapiro-Wilk-test, showing non-normal distribution. Hence data were evaluated for statistical significance using the non-parametric sign-test. Analyses were performed using SPSS for Windows, Version 15.0 (SPSS Inc., Chicago, IL, USA).

## Results

**Figure 1:**
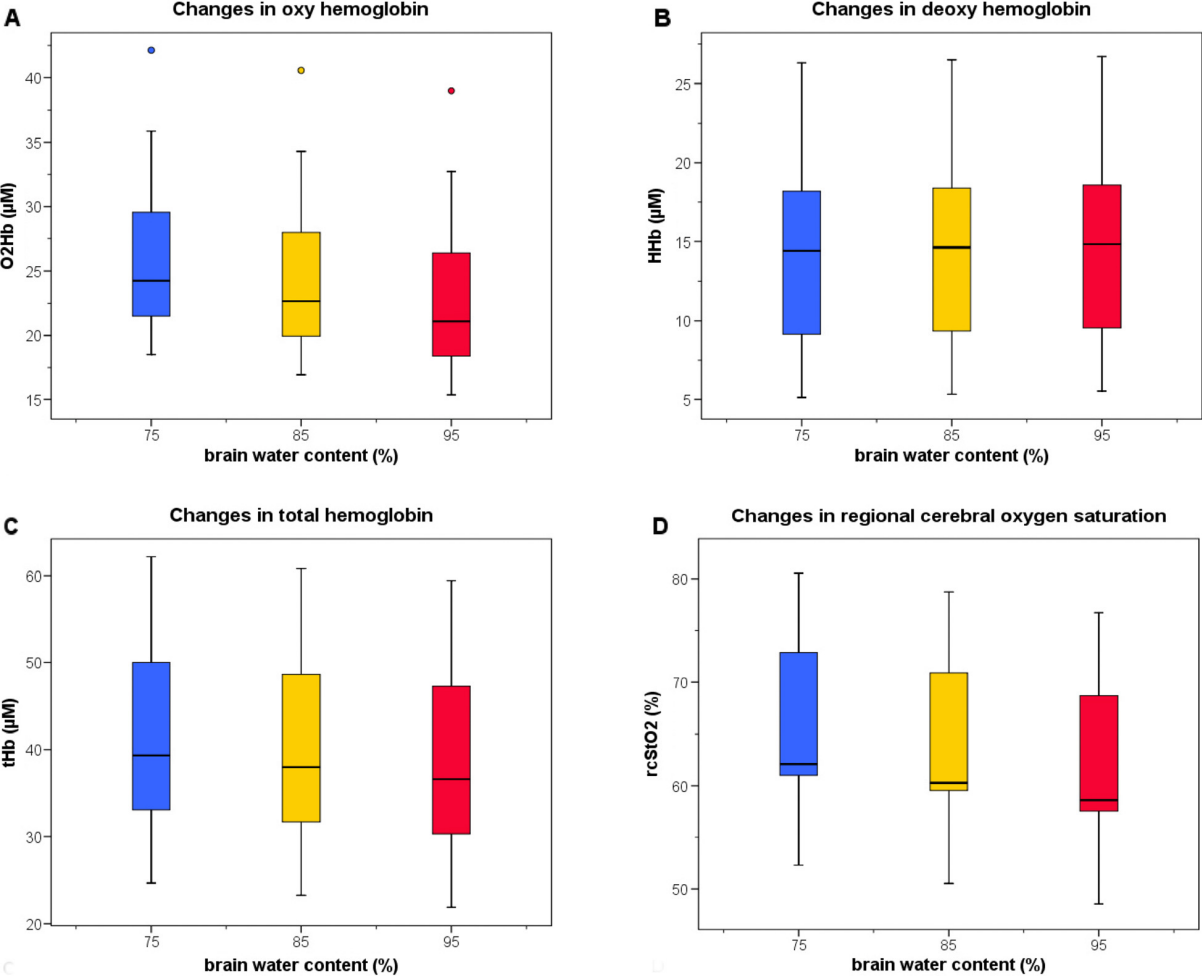
**(A-D): Calculated changes assuming different brain water contents.** O2Hb **(A)**, HHb **(B)**, tHb **(C)** and rcStO2 **(D)** calculated assuming different brain water contents of 75, 85 and 95%, respectively.

Different assumptions for BWC resulted in relevant changes in calculated concentrations of O_2_Hb and to a lesser extent of HHb. With the assumption of a higher BWC the computed concentration of O_2_Hb decreased and the computed concentration of HHb increased. Consequently, the tHb concentration and the rcStO_2_ decreased as shown in Figure 1. All comparisons between different assumptions for BWC yielded p-values < 0.0001 for all parameters evaluated. The computed values for O_2_Hb, HHb, tHb and rcStO_2_ at different BWC are shown in Table 2. Assuming BWC = 75% instead of BWC = 95% resulted in an overestimation of rcStO_2_ of 4 (1–8)%.In Figure 1 (A-D), oxygenated hemoglobin, deoxygenated hemoglobin, total hemoglobin and regional cerebral oxygen saturation are plotted against assumed BWCs.

**Table 2:** Hemoglobin concentrations and hemoglobin oxygen saturation for different assumptions of brain water content

	**BWC**	**Minimum**	**Median**	**Maximum**	**Q1**	**Q3**
O_2_Hb (μM)	75	18.51	24.24	42.13	21.34	32.18
	85	16.93	22.67	40.56	19.77	30.61
	95	15.36	21.10	38.99	18.20	29.04
HHb (μM)	75	5.13	14.43	26.30	8.67	19.04
	85	5.34	14.63	26.50	8.87	19.24
	95	5.54	14.83	26.71	9.07	19.44
tHb (μM)	75	24.63	39.36	62.17	31.64	51.45
	85	23.26	37.99	60.80	30.26	50.08
	95	21.89	36.62	59.43	28.89	48.71
rcStO_2_ (%)	75	52	62	81	59	76
	85	51	60	79	58	74
	95	49	59	77	56	72
Q1 = lower quartile;						
Q3 = upper quartile						

## Discussion

NIRS is increasingly used in neonatal intensive care. Instruments suitable and approved for continuous monitoring in this age group give readings on a measure of cerebral oxygenation based on several assumptions that may not hold true in a given infant – and hence displayed values of cerebral oxygenation may not be appropriate in certain individuals.

Our study addressed the question whether the assumption of different BWCs consistently influences O_2_Hb, HHb, tHb and rcStO_2_ readings in a realistic sample of clinically stable preterm infants using computations based on absolute coefficients μ_a_ and μ_s_’ determined by multi-distance FD-NIRS and the diffusion equation for homogeneous, semi-infinite media.

A clinically relevant overestimation of “true” rcStO_2_ by up to 8% may result if BWC is incorrectly assumed to be only 75% in an extremely immature infant with a true BWC of 90-95%. This influence of different BWCs within the physiological range encountered in the neonatal intensive care unit on parameters of cerebral oxygenation is disregarded by most manufacturers of NIRS devices and also neglected by many clinicians who rely on readings of parameters of cerebral oxygenation for guiding cardiovascular therapy.

This systematic overestimation of rcStO_2_ by 4% (1%-8%), is probably not important in settings where rcStO_2_ trend monitoring is used e.g., during surgical interventions, and whenever relative changes of cerebral oxygenation in relation to a ‘normal’ baseline are observed to indicate cardiovascular interventions. Neonatal applications of cerebral oxygenation monitoring frequently lack a ‘normal baseline’ as rcStO_2_ monitoring is used in extremely preterm [7-9] and asphyxiated infants after resuscitation [10]. Furthermore, neonatal cerebral oxygenation monitoring is intended for days rather than just a few hours (e.g., [9]). As indicated in an European collaborative phase 2 trial of rcStO_2_-monitoring in extremely preterm infants, neonatologists are indeed interested in long-term continuous rcStO_2_ monitoring and, in the absence of a ‘normal baseline’, do rely on absolute rcStO_2_ readings [7]. Furthermore, suggested treatment algorithms indicate cardiovascular interventions if absolute cut-off values of rcStO_2_ are exceeded [8]. Whenever absolute readings of rcStO_2_ are relied upon for clinical decision making, a systematic over-/underestimation of rcStO_2_ may be of clinical importance.

For various reasons, including inhomogeneity of the tissue and issues of probe placement (underlying blood vessels, skin, background absorbers, different scattering properties, hair and texture, etc.), the signal-to-noise ratio is poorer and the limits of agreement after repeated repositioning of the NIRS-probe are greater in rcStO_2_-monitoring than in SaO_2_-monitoring using pulse oximetry (reviewed in [11]). Bland-Altman bias analyses revealing poor agreement with 95% limits of agreement of up to -17% to +17% have been reported previously [12]. More recently, using the ISS Oxiplex TS which was also used in this study, Arri et al. demonstrated that the test retest variability of rcStO_2_ measurements was approximately 5% for preterm infants [13], similar to test retest variability of 5% reported by Sorensen using the NIRO 300 [14]. Based on this more recent data, a systematic overestimation of rcStO_2_ by 4% (1%-8%) due to incorrect assumptions of BWC is considerable. Moreover, this systematic bias will add to the imprecision of the method and, in contrast to random factors, it is a systematic error that will not be overcome by averaging.

Our findings may also be of importance in the interpretation of longitudinal studies: Previously reported longitudinal data suggested that rcStO_2_ values decrease in preterm infants during the first 6 weeks of life despite stable cerebral blood flow index, which was interpreted as an increase in metabolic rate of oxygen [15]. In this study, rcStO_2_ was calculated based on the probably incorrect assumption of a constant BWC of 75% throughout the study period. The results of our simulation suggest that incorrect underestimation of BWC early on, may have contributed to the findings and that the postnatal decrease in rcStO2 and the increase in the metabolic rate of oxygen may have been overestimated.

It is obvious that smaller differences between assumed and actual BWC will result in smaller deviations of O_2_Hb, HHb, tHb, and rcStO_2_ from reality. In fact, depending on postmenstrual and postnatal age most BWC-values will range between 80% and 90% [6]. Furthermore, our data are only applicable to the wavelengths used herein. Different wavelengths with different ratios between the extinction coefficients for O_2_Hb, HHb and water will result in different degrees of deviation from reality if BWC is not taken into account. In general, the higher the extinction coefficient of water in relation to that of O_2_Hb or HHb at a given wavelength, the more relevant will be the impact of the difference between assumed and actual BWC.

Effects of different assumptions of BWC on rcStO_2_ readings of different devices will depend on the wave lengths used (as outlined above) and on the underlying algorithms for determination of rcStO_2_. In contrast to the instrument used for our study, unfortunately, many manufacturers of NIRS oximeters did not publish their algorithms and it is unknown how they deal with the water assumption.

We have previously described [16] that introducing a water term into equations describing the relation between the absorption coefficient, μa, and the slope of the decrease in light intensity using multi-distance FD-NIRS resulted in minor changes in StO_2_-measurements of the neonatal head if a constant BWC of 90% was assumed. However, this introduction of a water term resulted in large changes (absolute change in StO_2_ of up to 18% or relative change up to 30%) if the water content was assumed to be 70% in StO_2_-measurements on the adult arm. The present data complement our previous results, accounting for different assumptions for BWC in the range encountered between extremely preterm infants and early childhood. Those different assumptions for BWC will systematically bias results of HHb, O_2_Hb, tHb and StO_2_ measurements, overestimating StO_2_ if too low BWC is assumed. Although the median bias introduced by incorrect assumptions of BWC may be small (Table 2), in the occasional infant overestimation of StO_2_ may be clinically relevant.

Developmental changes in BWC should be considered in the clinical setting, especially in preterm infants, because a median difference in rcStO_2_ of 4% and a difference in rcStO_2_ of up to 8% in individual patients could change therapeutic decisions with potential long-term consequences.

## Conclusion

Changing assumptions of BWC resulted in systematic modifications of computed O_2_Hb and HHb and in consecutive clinically relevant changes in rcStO_2_ of up to 8%. Disregarding maturational changes in BWC is another factor contributing to inadequate accuracy of absolute measures of cerebral oxygenation by standard NIRS devices. Neonatologists should be aware of the fact that rcStO_2_ will be overestimated by up to 8% if algorithms for calculating the measure of cerebral oxygenation are based on adult BWC.

## Abbreviations

FD-NIRS: Frequency-Domain Near-Infrared Spectroscopy; μa: Absorption coefficient; μs’: Reduced scattering coefficient; rcStO2: Regional cerebral oxygen saturation; HHb: Deoxygenated hemoglobin; O_2_Hb: Oxygenated hemoglobin; tHb: Total hemoglobin; BWC: Brain water content.

## Competing interests

The authors declare that they have no competing interests.

## Authors’ contributions

AD designed the study and performed all NIRS-measurements, ran the data collection, performed the analysis and drafted the initial manuscript and revised the manuscript; MW advised NIRS aspects of the study and provided important advice for the calculations, reviewed and revised the manuscript making important intellectual contributions; CFP supervised the project as the head of department and reviewed and revised the manuscript making important intellectual contributions; ARF was co-coordinator of the project, supervised data analyses and reviewed and revised the manuscript making important intellectual contributions. All authors read and approved the final manuscript.

## Pre-publication history

The pre-publication history for this paper can be accessed here:

http://www.biomedcentral.com/1471-2431/14/206/prepub
